# Robotic versus Open Pancreatoduodenectomy for Pancreatic and Periampullary Tumors (PORTAL): a study protocol for a multicenter phase III non-inferiority randomized controlled trial

**DOI:** 10.1186/s13063-021-05939-6

**Published:** 2021-12-27

**Authors:** Jiabin Jin, Yusheng Shi, Mengmin Chen, Jianfeng Qian, Kai Qin, Zhen Wang, Wei Chen, Weiwei Jin, Fengchun Lu, Zheyong Li, Zehua Wu, Li Jian, Bing Han, Xiao Liang, Chuandong Sun, Zheng Wu, Yiping Mou, Xiaoyu Yin, Heguang Huang, Hao Chen, Georgios Gemenetzis, Xiaxing Deng, Chenghong Peng, Baiyong Shen

**Affiliations:** 1 Department of Pancreatic Surgery, Ruijin Hospital, Shanghai Jiao Tong University School of Medicine, Shanghai, China; 2 Department of Hepatobiliary Surgery, The First Affiliated Hospital of Xi’an Jiaotong University, Xi’an, Shanxi Province China; 3 Department of Pancreaticobiliary Surgery, The First Affiliated Hospital, Sun Yat-sen University, Guangzhou, Guangdong Province China; 4 Department of Gastroenterology and Pancreatic Surgery, Zhejiang Provincial People’s Hospital, Hangzhou, Zhejiang Province China; 5 Department of General Surgery, Fujian Medical University Union Hospital, Fuzhou, Fujian Province China; 6 Department of General Surgery, Sir Run Run Shaw Hospital, Zhejiang University School of Medicine, Hangzhou, Zhejiang Province China; 7 Department of Hepatobiliary and Pancreatic Surgery, The Affiliated Hospital of Qingdao University, Qingdao, Shandong Province China; 8 Clinical Research Center, Shanghai Jiao Tong University School of Medicine, Shanghai, China; 9 Department of Hepatopancreatobiliary and Transplant Surgery, Royal Infirmary Edinburgh, Edinburgh, UK; 10 Department of Pancreatobiliary Surgery, Glasgow Royal Infirmary, Glasgow, UK

**Keywords:** Robotic, Robot-assisted, Minimally invasive, Pancreatoduodenectomy, Whipple, Pancreatic cancer, Outcomes, Recurrence, Survival

## Abstract

**Background:**

Pancreatoduodenectomy is a complex and challenging procedure that requires meticulous tissue dissection and proficient suturing skills. Minimally invasive surgery with the utilization of robotic platforms has demonstrated advantages in perioperative patient outcomes in retrospective studies. The development of robotic pancreatoduodenectomy (RPD) in specific has progressed significantly, since first reported in 2003, and high-volume centers in pancreatic surgery are reporting large patient series with improved pain management and reduced length of stay. However, prospective studies to assess objectively the feasibility and safety of RPD compared to open pancreatoduodenectomy (OPD) are currently lacking.

**Methods/design:**

The PORTAL trial is a multicenter randomized controlled, patient-blinded, parallel-group, phase III non-inferiority trial performed in seven high-volume centers for pancreatic and robotic surgery in China (> 20 RPD and > 100 OPD annually in each participating center). The trial is designed to enroll and randomly assign 244 patients with an indication for elective pancreatoduodenectomy for malignant periampullary and pancreatic lesions, as well as premalignant and symptomatic benign periampullary and pancreatic disease. The primary outcome is time to functional recovery postoperatively, measured in days. Secondary outcomes include postoperative morbidity and mortality, as well as perioperative costs. A sub-cohort of 128 patients with pancreatic adenocarcinoma (PDAC) will also be compared to assess the percentage of patients who undergo postoperative adjuvant chemotherapy within 8 weeks, in each arm. Secondary outcomes in this cohort will include patterns of disease recurrence, recurrence-free survival, and overall survival.

**Discussion:**

The PORTAL trial is designed to assess the feasibility and safety of RPD compared to OPD, in terms of functional recovery as described previously. Additionally, this trial will explore whether RPD allows increased access to postoperative adjuvant chemotherapy, in a sub-cohort of patients with PDAC.

**Trial registration:**

ClinicalTrials.govNCT04400357. Registered on May 22, 2020

**Supplementary Information:**

The online version contains supplementary material available at 10.1186/s13063-021-05939-6.

## Background

Pancreatoduodenectomy (PD) is the surgical approach of choice for benign and malignant tumors of the pancreatic head and periampullary region, and it is considered the only potential curative treatment option [1]. It is a sophisticated operation that requires proficient surgical skills, both for the dissection of the tumor from the adjacent vascular structures and for the restoration of gastrointestinal tract continuity with the performance of the pancreatic, biliary, and gastric anastomosis. This level of surgical complexity may result in significant postoperative morbidity, and data suggest that surgeon experience [2] and hospital volume [3] are critical for the achievement of favorable outcomes in pancreatic surgery. The introduction of minimally invasive surgery (MIS) promptly led to the first report of a laparoscopic pancreatoduodenectomy (LPD) [4]; however, broader adoption of this technique was relatively slow due to multiple parameters, which include limited instrument motion range, deficient ergonomics, and a long learning curve [5]. A recent phase 2/3 randomized controlled trial comparing LPD and open pancreatoduodenectomy (OPD) was terminated early, due to increased mortality in the LPD arm [6], demonstrating further the technical limitations of the technique, as well as the importance of substantial surgeon experience in minimally invasive pancreatic surgery (MIPS).

Utilization of the daVinci® robotic surgical platform (dVSS, Intuitive Surgical, Sunnyvale, CA, USA) has demonstrated an advantage compared to laparoscopic instruments, mainly due to improved three-dimensional visualization of the surgical field, tremor reduction, and additional instrument degrees of freedom [7]. As a result, robotic pancreatic surgery has gained popularity in recent years. In robotic pancreatoduodenectomy (RPD), specifically complication rates, including postoperative pancreatic fistula (POPF), delayed gastric emptying (DGE), and bleeding [8, 9], are comparable to OPD, after surpassing the learning curve [10, 11]. Additionally, estimated intraoperative blood loss, postoperative pain, and length of stay appear to be improved in RPD [12, 13]. Yet, the significant costs of the robotic platform and the lack of robust evidence to demonstrate meaningful benefit have also raised concerns in the surgical community regarding its generalized acceptance [14].

The integration of robotic pancreatic surgery in China was initiated in high-volume centers for patients with pancreatic disease, early in the development of MIS. This process allowed for optimal identification of the learning curve, assessment, and development of training, and deeper integration of the robotic surgical approach for the management of patients with pancreatic disease [11]. Since most clinical evidence for robotic surgery is based on single-center retrospective case series and non-randomized data, the need for a randomized controlled trial to objectively assess the feasibility and patient outcomes in RPD is evident, as indicated by the recent consensus guidelines on MIPS [15]. This report describes the study design and protocol of the PORTAL trial, a non-inferiority multicenter randomized controlled trial, conducted in seven national high-volume centers for pancreatic and robotic surgery. The primary goals of this trial are to prospectively [1] assess and compare the time from surgery to functional recovery between patients who undergo OPD versus RPD and [2] investigate the percentage of patients who initiate postoperative adjuvant chemotherapy within 8 weeks from surgery in a sub-cohort of patients with pancreatic adenocarcinoma.

## Methods

### Trial design

The PORTAL trial is a multicenter randomized controlled, patient-blinded, parallel-group, phase III non-inferiority trial performed in seven high-volume centers for pancreatic and robotic surgery in China (> 20 RPD and > 100 OPD annually in each participating center). Eligible patients for enrollment will be randomized in a 1:1 ratio to one of the two trial arms: robotic pancreatoduodenectomy and open pancreatoduodenectomy.

### Patient population

All patients who present in the participating centers with an indication for elective pancreatoduodenectomy will be assessed for eligibility regarding trial participation. Clinical indications for the procedure will include malignant periampullary and pancreatic lesions (pancreatic adenocarcinoma, distal cholangiocarcinoma, duodenal and ampullary carcinoma), as well as premalignant and symptomatic benign periampullary and pancreatic disease (pancreatic neuroendocrine tumors, cystic lesions, and chronic pancreatitis). Eligible patients will be assessed and informed by participating registered members of the surgical teams involved in the trial at the outpatient clinics.

### Inclusion and exclusion criteria

The inclusion criteria for trial eligibility are [1] age ≥ 18 years [2]; clinical indication for pancreatoduodenectomy [3]; patient eligibility for both RPD and OPD [4]; patient performance status of 0 or 1 based on comorbidities, assessed using the Karnofsky score and the Nutritional Risk Screening Tool (NRS-2002) score; and [5] imaging with computed tomography (CT) and/or MRI pancreas performed within 8 weeks for benign and 4 weeks for malignant disease; for the latter, tumor staging with MRI liver, CT chest, and cancer antigen 19-9 (CA19-9) is also mandatory not earlier than 4 weeks prior to surgery.

The exclusion criteria are [1] body mass index > 35 kg/m^2^, [2] pregnancy, [3] previous history of major abdominal surgery, [4] requirement for multivisceral resection (additional surgical resection of surrounding solid organs), [5] ongoing treatment for other primary malignancy, [6] preoperative chemotherapy and/or radiotherapy in patients with pancreatic adenocarcinoma, and [7] involvement of major regional vessels by the tumor (portal and superior mesenteric vein, superior mesenteric artery, hepatic artery, celiac artery) as defined by the borderline resectable and locally advanced nomenclature [16].

### Randomization process (Fig. 1)

Eligible patients will be identified and discussed about the trial at the time of initial outpatient clinic assessment. Details will be provided to the patients about the goals and the structure of the trial and how it will affect the treatment approach. Recruited patients will be informed further in detail and will sign a written informed consent prior to treatment initiation, which can be revoked at any time. After signing the informed consent, all included patients will be randomized in a 1:1 ratio between robotic and open pancreatoduodenectomy using an online randomization module (IRT, Shanhu Health, Shanghai, China). Eligible patients will be variably randomized in equal proportions, with variable sizes blocks. After enrollment, they will be randomly stratified preoperatively based on [1] risk of developing a postoperative pancreatic fistula (POPF) using an externally validated model [17]: high risk (pancreatic duct diameter < 3 mm on preoperative CT at the pancreatic neck, and/or body mass index (BMI) > 25 kg/m^2^) versus low risk (pancreatic duct diameter ≥ 3 mm on preoperative CT at the pancreatic neck, and/or BMI ≤ 25 kg/m^2^) [2]; presence of endoscopic stenting for preoperative biliary drainage; and [3] diagnosis of pancreatic adenocarcinoma. Patient randomization and allocation between the two arms is performed with dedicated software (irtlite.irtone.com); after allocation, the patient details and allocated arm are typed into sealed envelopes by the randomization team, and the clinical nurse specialists deliver the envelopes to the appropriate surgical teams. The envelopes will be opaque and numbered with the patient trial ID number and will be opened by the responsible surgical team 1 day prior to surgery. Participating patients will be blinded to the procedure until postoperative day 5 or until functional recovery is achieved. Blinding will occur with the placement of identical wound dressings in all patients: on the presumed midline or J-incision and the placed trocars sites and extraction site incision in the RPD group and on the midline or J-incision and the presumed trocar sites and extraction site incision in the OPD group (Fig. 2a). Wound changes will occur as clinically indicated in an attempt to maintain patient blinding. This is a feasible approach that has been tested in previous randomized prospective trials [6, 18]. Unblinding of participating patients will be available as per their request; if a patient is accidentally unblinded, the event will be noted, and the patient will be excluded from the per-protocol analysis.

**Fig. 1:**
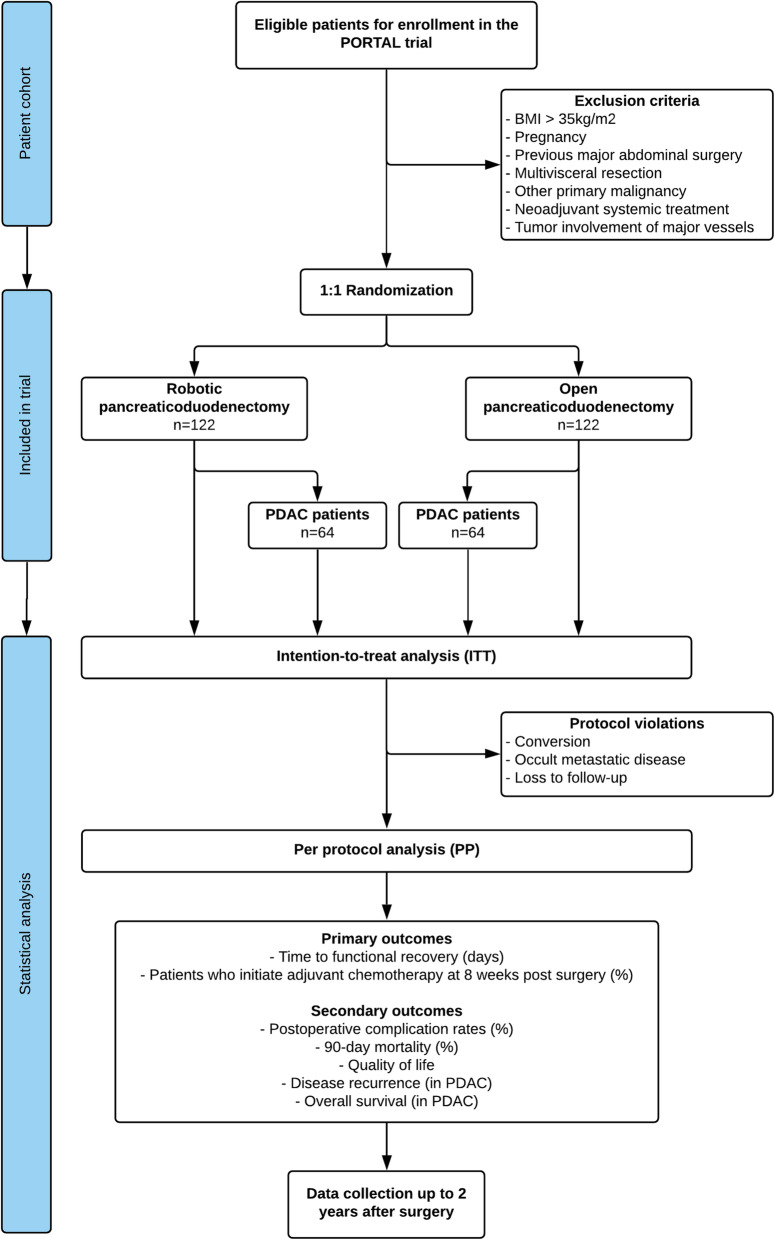
PORTAL trial flowchart: patient inclusion and randomization, arms design, and statistical analysis. BMI, body mass index; PDAC, pancreatic adenocarcinoma; ITT, intention-to-treat; PP, per-protocol

**Fig. 2:**
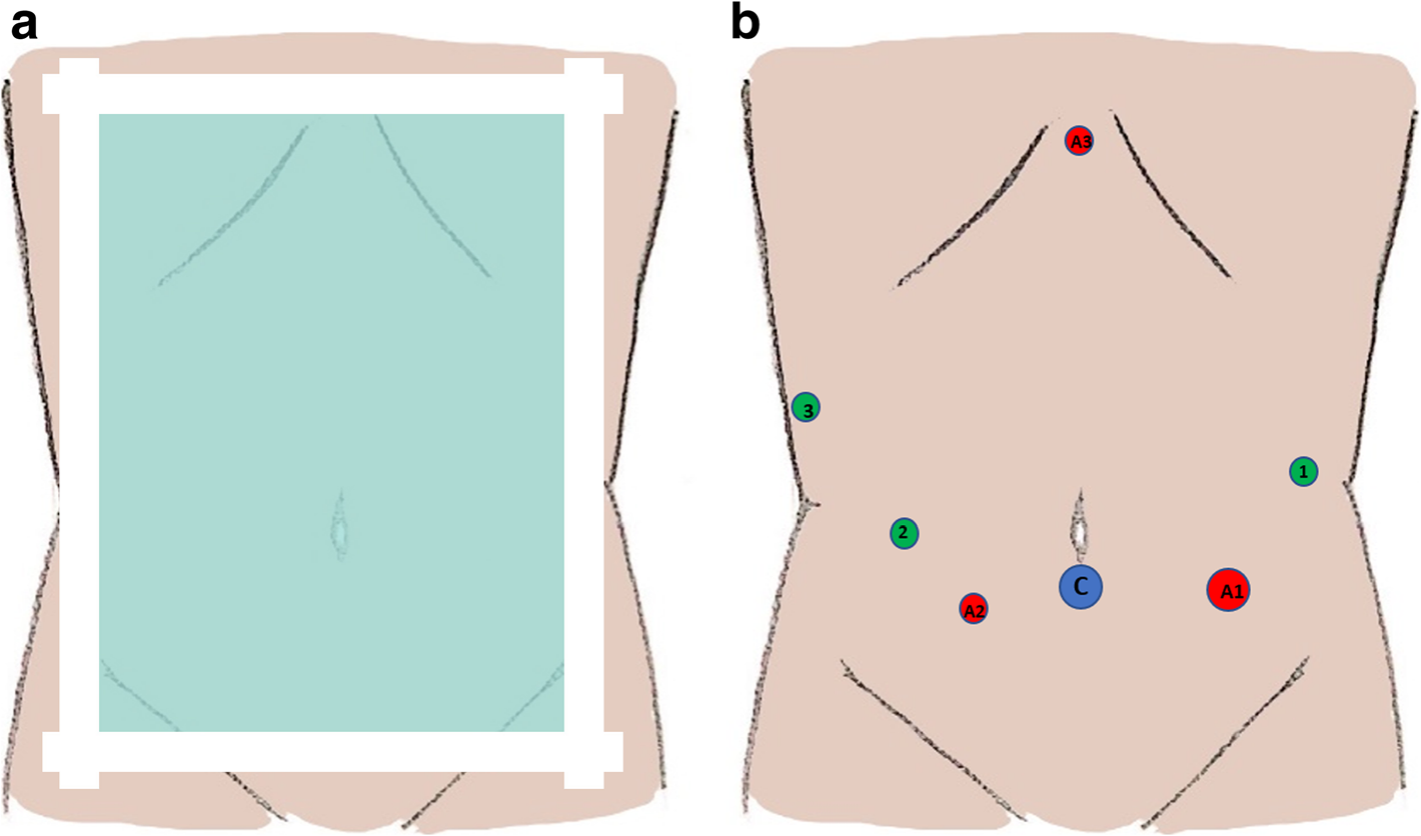
**a** Illustration of patient blinding in the postoperative setting; all wound dressings and drain sites are identical in patients who undergo both open and robotic pancreatoduodenectomy. **b** Illustration of trocar placement in robotic pancreatoduodenectomy; 1–3, robotic ports. C, camera port, A1––A3: assisting laparoscopic ports (utilized on individual patients basis)

### Operative technique: robotic pancreatoduodenectomy

The daVinci® Si or Xi Surgical System will be utilized for patients randomized to the RPD arm. The basic steps of the procedure are as follows: In the immediate preoperative setting, prior to anesthesia induction, subcutaneous deep venous thromboprophylaxis and intravenous antibiotics are administered, and the patient is placed in a supine position with split legs and the left arm in 90° abduction. After prepping and draping, access to the abdominal cavity is established with a supraumbilical or infraumbilical port, which accommodates the camera; the rest of the trocars are placed as follows (Fig. 2b): three robotic arm ports are placed in the left and right upper quadrant midclavicular line and the right subcostal anterior axillary line. A laparoscopic assistant port is also placed in the lower left quadrant midclavicular line; additional ports for further laparoscopic assistance and placement of liver retractor can also be utilized. The table is turned left at approximately 15° and the patient is placed in reverse Trendelenburg position to accommodate the docking of the robot.

A diagnostic laparoscopy is initially performed to exclude occult abdominal metastatic disease. Tissue dissection is initiated with access in the lesser sac by cutting the gastrocolic ligament, followed by mobilization of the hepatic flexure of the colon and performance of a Kocher maneuver for mobilization and exposure of the pancreatic head. Identification of the avascular plane between the posterior surface of the pancreatic head and the underlying inferior vena cava and right kidney follows. The fourth part of the duodenum is mobilized, and 40–50 cm of the jejunum is pulled through the gap under the mesenteric root and transected 10 cm from the ligament of Treitz. The inferior border of the pancreas is further dissected, the superior mesenteric vein (SMV) is visualized, and ligation of the gastrocolic trunk is performed. Attention is then turned to the superior pancreatic border, where the stomach is transected immediately proximal to the pylorus using a surgical stapler. The 8a lymph node is resected and sent to pathology, and posteriorly, the gastroduodenal (GDA) and right gastric arteries are also visualized and transected between Hem-o-lok clips (Teleflex Medical, Research Triangle Park, NC, USA). The anterior surface of the pancreas is exposed, and a tunnel is created under it; the pancreatic parenchyma is transected at the neck with diathermy or harmonic scalpel and sharply at the pancreatic duct. If indicated, frozen biopsies from the transected pancreatic margin are sent to pathology, and a 6-Fr catheter is placed in the pancreatic duct to secure visualization. The gallbladder is detached from the gallbladder fossa, and the hepatoduodenal ligament is then dissected to identify the common hepatic duct; this is transected above the junction with the cystic duct, close to the confluence. The specimen is positioned to the right of the PV/SMV for optimal exposure. The uncinate process is further mobilized with ligation of the inferior pancreatoduodenal arteries and veins, and the specimen is freed and placed in a collection bag, which is left temporarily within the abdomen.

The reconstruction phase starts with a single jejunal loop being passed in a retrocolic fashion through a small incision in the mesocolon on the right of the mesocolic vessels or through the tunnel under the mesenteric root. The pancreaticojejunostomy (PJ) is performed in an end-to-side fashion as a double-layer duct-to-mucosa anastomosis with interrupted 3/0 and 5/0 or 6/0 Prolene sutures for the outer and inner layer, respectively. Ten centimeters distally on the same jejunal loop, the hepaticojejunostomy (HJ) is constructed with running or interrupted 4/0 or 5/0 PDS or Prolene sutures, based on the diameter of the common hepatic duct. Lastly, the gastrojejunostomy is performed with single-layer running 3/0 V-loc (Covidien, New Haven, CT, USA) or 3/0 Vicryl sutures, or with an endostapler. If a pylorus-preserving pancreatoduodenectomy (PPPD) is decided, the subsequent duodenojejunostomy is performed in a similar fashion to the GJ. The abdomen is thoroughly inspected, and hemostasis is secured and two large-bore double-lumen surgical drains are placed in proximity to the PJ and the HJ. The specimen is extracted via the extended periumbilical trocar incision or a Pfannenstiel incision, which is closed in layers, and all trocars are removed, and skin incisions are sutured. Placement of wound catheters for postoperative pain control is optional.

Minor variations in the robotic surgical approach based on individual surgeon experience and preference, especially regarding the pancreatic and/or biliary anastomotic technique, will be discussed and accepted accordingly; documentation of any variations in patient records will be mandatory. Conversion rates from the robotic to the open procedure will be recorded in detail and assessed based on urgent and non-urgent conditions. Patients assigned in the robotic arm, who undergo a conversion procedure will be included as part of the robotic group in an intention-to-treat analysis.

### Operative technique: open pancreatoduodenectomy

In the control arm, patients will undergo an open pancreatoduodenectomy, which is the standard of care and has a long-standing safety record. A midline or right J-shaped subcostal incision is performed for access in the abdominal cavity. The operative steps are similar to the robotic approach in terms of tumor dissection, specimen removal, and anastomotic reconstruction. Since OPD volumes in participating centers are very high with quality results, wider surgical variations from individual surgeons are anticipated. These variations will be evaluated in advance and allowed accordingly, and they will also be documented in detail in patient records.

### Postoperative patient care

All participating centers are following a modified enhanced recovery after surgery (ERAS®) protocol, which focuses mainly on patient mobilization, pain management, and patient-directed oral intake and includes the following landmarks: removal of NGT on postoperative day and patient mobilization (POD) 1, clear fluids on POD 2, free fluids on POD4, and diet on POD5 as tolerated. Medical discharge will occur as a joint decision of the surgical team and the local community services that may need to be involved in the patient’s care as an outpatient. All patients are essentially declared fit for discharge as soon as functional recovery is achieved.

### Primary outcome measures

The primary outcome measure of the PORTAL trial for all participating patients is time to postoperative functional recovery, measured in days. The patients’ postoperative course will be evaluated daily by the nursing and medical staff and will be communicated to the study coordinators for documentation. Postoperative criteria that need to be fulfilled independently to achieve functional recovery status are as previously described [18]:

- Discontinuation of intravenous or subcutaneous analgesia and preservation of adequate control levels (assessed by a pain score).
- Ability to maintain sufficient oral caloric intake as defined by the institutional dietetic services (no less than 50% of recommended dietary intake), without intravenous fluid support.
- Restoration of patient ambulation to an independent or preoperative level if mobility deficits were present.
- No clinical or biochemical signs of ongoing abdominal infection affecting the patient’s performance status: absence of fever and normalization of inflammatory markers (white cell count, procalcitonin, and/or C-reactive protein). Patients can be in oral antibiotics.

Additionally, in a sub-cohort of patients with pancreatic ductal adenocarcinoma assessed between the two study arms, the primary outcome will be the percentage of patients who achieve administration of adjuvant chemotherapy at 8 weeks postoperatively. The chemotherapy regimen will be decided by the oncology service on an individual basis and will not affect the comparison.

### Secondary outcome measures (Tables 1 and 2)

Multiple secondary outcomes will be compared within the trial. Intraoperative parameters will include operative time (from the first incision to skin closure) and estimated blood loss. Additionally, pathologic characteristics will also be studied, including tumor size and degree of differentiation, presence of perineural and/or lymphovascular invasion in PDAC, resection margin status, and number of harvested and infiltrated regional lymph nodes, as defined by the 8th edition of the American Joint Committee for Cancer [19]. Regarding the resection margin (R), it will be defined as R0 when tumor cells are detected > 1 mm from the margin, R1 when they are detected ≤ 1 mm from the margin, and R2 when there is macroscopically margin positivity. In the postoperative setting, the main secondary outcomes are complication rates as defined by the Clavien-Dindo classification [20] within 30 days and mortality within 90 days from operation. The trial will assess the occurrence of postoperative pancreatic fistula, biliary leak, chyle leak, delayed gastric emptying, bleeding, and surgical site infections (SSI) in both arms as defined by the International Study Group on Pancreatic Surgery [21–24] and the International Study Group in Liver Surgery [25], with a specific focus on the severe complications (Clavien-Dindo grade IIIa and higher). Subsequently, re-intervention (radiological, endoscopic, or surgical) and readmission rates will also be assessed. All interventions necessary for the treatment of participating patients in terms of management of potential complications are allowed. There are no limitations regarding patients’ care within this trial. All performed interventions will be documented in detail and assessed accordingly. Perioperative costs including postoperative hospitalization and readmissions within 30 days from the surgery will also be documented and evaluated. Lastly, quality of life (QoL) measurements will occur using patient questionnaires up to 3 months after surgery.

**Table 1:** Trial progression portrayed as per Standard Protocol Items: Recommendation for Interventional Trials (SPIRIT) guidelines

Event	Preoperative setting (3 to 14 days pre-op)	Day of surgery	POD 1	POD 2	POD 3	POD 5	POD8/discharge day	POD 14	POD 30	POD 90	6 months post-op	1 year post-op	2 years post-op/time of death
Eligibility screening	X												
Informed consent	X												
Baseline characteristics	X												
Arm allocation	X												
QoL questionnaire (EQ-5D-5 L)	X						X	X	X	X	X	X	
Intraoperative outcomes		X											
Postoperative outcomes/secondary endpoints			X	X	X	X	X	X	X	X			
Primary endpoint assessment							X			X			
Recurrence-free and overall survival assessment													X

*pre-op* preoperative, *post-op* postoperative, *POD* postoperative day, *QoL* quality of life

**Table 2:** Primary and secondary outcomes of the PORTAL trial

***Variable***	
**Primary outcomes** Time to functional recovery, *n* (days) Patients with PDAC who start adjuvant chemotherapy at 8 weeks, *n* (%)	
**Secondary outcomes** Estimated blood loss, ml Operative time, min Length of hospital stay, days 30-day morbidity, *n* (%) < Clavien-Dindo grade IIIa ≥ Clavien-Dindo grade IIIb 30-day mortality, *n* (%) 90-day mortality, *n* (%) 30-day readmission, *n* (%) Reoperation, *n* (%) Quality of life assessment Perioperative costs, $ In PDAC Resection margin status, *n* (%) Harvested lymph nodes, *n* Positive lymph nodes, *n* Recurrence, *n* (%) Recurrence site, *n* (%) Recurrence-free survival, month*s* Overall survival, months	

*OPD* open pancreaticoduodenectomy, *RPD* robotic pancreaticoduodenectomy, *PDAC* pancreatic adenocarcinoma

### Analysis of long-term outcomes in patients with pancreatic adenocarcinoma

The sub-cohort of patients with PDAC in the two arms will be followed up for a period of 2 years after surgery, or until their time of death within this period. During that time, the administration and type of systemic therapy will be documented; the main focus will be the recurrence-free survival (RFS) and overall survival (OS), defined as the time from surgery to the first radiological evidence of disease recurrence and death, respectively.

### Data collection and patient follow-up

**Fig. 3:**
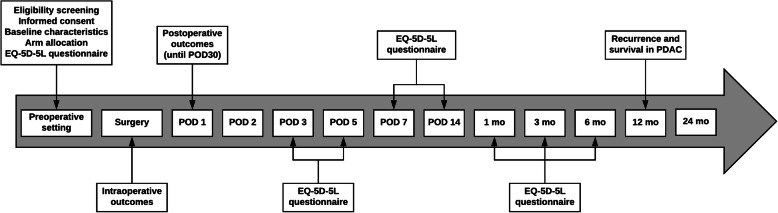
Timeline diagram of the studied primary and secondary outcomes. EQ-5D-5L, Quality of Life Questionnaire; POD, postoperative day; PDAC, pancreatic adenocarcinoma

At the time of patient consent for participation in the trial and prior to randomization, baseline patient characteristics will be recorded. These will mainly comprise demographics (age, sex, BMI) and past medical history, including comorbidities for the assessment of performance status. Clinical presentation will also be documented with a focus on patient symptoms and signs; in cases of solid tumors, imaging data will also be collected and evaluated: tumor size and staging, vessel involvement, and pancreatic duct (PD) diameter, as well as the presence of preoperative biliary drainage and treatment with somatostatin analogs. At the time of surgery, the pancreatic texture will be documented (soft versus hard gland) and the PD diameter will be also measured. Previously discussed minor variations in operative approach and anastomotic reconstruction will be added in patient record forms. Regarding the quality of life assessment, patients will complete the latest translated version of the EuroQoL EQ-5D-5 L questionnaire, which is a self-reported assay focusing on five parameters: pain/discomfort, anxiety/depression, usual activities, self-care, and mobility. Each of these parameters is scored from 0 to 5 (no issue, slight problem, moderate problem, severe problem, unable). Patients also score independently their health status on a scale numbered from 0 to 100: 100 meaning the best health they can imagine, and 0 the worst. The questionnaires will be filled and collected by medical or nursing staff on postoperative days 1, 3, 5, and 7 during the patients’ hospital stay; further questionnaires will be completed in the postoperative setting every week until day 30, every month until day 90, and every 3 months until 12 months, when the evaluation will terminate (Fig. 3). Remote completion will take place via telephone communication, by mail, or face-to-face for all patients who are followed up at the participating centers. Furthermore, in the postoperative setting, primary and secondary outcomes during the hospital stay will be documented daily by the trial coordinators. Patients will be further followed up after discharge with out-patient clinic appointments or via telephone/video conference as an alternative to monitor for delayed postoperative complications, quality of life changes, and disease recurrence patterns. Frequent communication and close monitoring of enrolled patients by the participating centers via telephone and mail will aim to promote patient retention during the trial period. All collected data are de-identified and stored safely in a centralized online database, which is accessible by all participating institutions for data input. The stored data are not available to individual collaborators and are only screened by two dedicated members of the trial team for assessment of wrong or missing data. Data processing regarding the results is conducted by a group of two independent statisticians who work under the data monitoring committee.

### Statistical considerations and analysis

The main hypothesis in the PORTAL trial is that functional recovery of patients who undergo RPD is non-inferior to the one of patients who undergo OPD. Based on institutional historical data and information from retrospective series in the literature, the time to functional recovery in OPD is 10 days with a standard deviation of 2.5 days. Non-inferiority of functional recovery in RPD will be assessed with a non-inferiority (*δ*) margin of 10% or 1 day. With a one-sided test, a significance level of 5% (*α* = 0.05), and a power (1 − *β*) of 80%, the cohort for the trial is calculated to 200 patients (per-protocol (PP) group), using the PASS 11.0 software (NCSS, Kaysville, UT, USA) with a sample allocation ratio of 1:1 between the RPD and OPD groups. However, we anticipate a conversion rate in the RPD group of 10%, 3% incidence of occult abdominal metastatic disease in both groups, and 5% of patients to be lost to follow-up. Therefore, the final cohort will include 122 patients in each arm (intention-to-treat (ITT), Fig. 1). The main analysis will be conducted on the ITT group according to the randomized initial assignment of patients in each study arm at baseline. All enrolled patients from the ITT population who do not fulfill the aforementioned protocol violations will be included in the PP group. Additionally, patient characteristics of those who will be excluded from the PP analysis will be separately evaluated. Non-inferiority will be concluded if the lower bound of the 95% confidence interval (CI) for the difference between the two groups in time to functional recovery is not lower than − 1 day of the non-inferiority margin.

A second primary endpoint of the study is the percentage of patients with a diagnosis of pancreatic adenocarcinoma in each study arm, who will be fit for the initiation of postoperative adjuvant chemotherapy 8 weeks after the day of surgery. Institutional empirical data demonstrate that 50% and 75% of patients who undergo OPD and RPD, respectively, are eligible for adjuvant chemotherapy at 8 weeks, which is considered the optimal time for postoperative systemic treatment. With the same power and significance level as in the ITT group, 64 patients with pancreatic adenocarcinoma will be included in each arm to assess for the superiority of RPD.

Primary and secondary outcome data for both ITT and PP groups will be compared utilizing appropriate statistical methodology. Categorical variables will be represented as frequency and proportion and will be analyzed using Fisher’s exact or chi-square tests, as appropriate. Continuous variables will be presented as mean values with standard deviation (SD), or median values with interquartile range (IQR), and will be compared using the two-sample *t*-test or Mann-Whitney *U* test, as per the type of distribution. A logistic regression analysis was performed for consideration of randomization stratification, based on the predetermined parameters. Survival of patients with pancreatic adenocarcinoma between the two arms will be estimated using the Kaplan-Meier analysis method and compared between the groups with the log-rank test. The SAS software v. 9.2 (SAS Institute Inc., USA) was utilized for all statistical analyses.

### Trial monitoring: quality and safety plan

Participating surgeons in the PORTAL trial have significant experience in both open and robotic pancreatic resections: all have at least 10 years of very high-volume open pancreatoduodenectomy (> 100 annually), have significant experience in laparoscopic pancreatectomy, and have implemented a robotic approach in pancreatic resections for more than 5 years. Regarding RPD, all participants in the trial have undergone formal robotic training via proctoring, official daVinci® training course, and video training and have subsequently performed > 30 RPD each. Therefore, they have passed the learning curve for RPD [10], and reported outcomes on mortality and morbidity are in line with the literature and comparable with the open approach. All procedures are also performed in very high-volume centers for pancreatic surgery (> 100 open and > 20 robotic cases annually). Cumulatively, all participating centers have performed approximately 1000 RPD. Furthermore, for safety monitoring purposes, all operations will be recorded and reviewed in retrospect to assess for technique optimization and evaluate major incidents that have led to conversion and/or significant patient morbidity. The safety of the robotic approach will be evaluated in standardized intervals by the appointed institutional ethics and data monitoring committees, which will advise regarding the trial’s status. More specifically, 30-day mortality will be monitored independently in each arm, and if a safety threshold of 5% is reached at any given time, the trial will be withheld for further safety evaluation and potential decision for early termination. If deemed necessary, major protocol modifications will be approved by the ethics committee and will be communicated to participating centers.

### Financial considerations

Patients enrolled in the PORTAL trial will receive no compensation for their participation. Additionally, they will not need to cover medical costs applied for randomization in the RPD arm. Costs of the robotic equipment will be covered cooperatively by the patients’ insurance companies and the trial sponsors. This trial is partially funded by Intuitive Surgical, which explicitly has no role in the study design and/or data collection and interpretation.

## Discussion

The main goal of the PORTAL trial is to prospectively assess the feasibility and safety of RPD in patients with benign and malignant lesions of the pancreatic head and periampullary region and further compare it with OPD. This protocol describes the study design and presents the analyses that will be published after the conclusion of the trial. The comparison/control group of OPD was selected because it is the standard of surgical practice and has a proven safety record when performed in high-volume centers.

The feasibility and safety of RPD have so far been exhibited only in retrospective series of high-volume institutions. Zureikat et al. initially reported comparable 30-day morbidity and 90-day mortality rates between RPD and OPD [12], and similar results were also shown in recent meta-analyses [26, 27]. Moreover, data from Ruijin Hospital, the leading institution in the PORTAL trial, similarly demonstrated a 2.1% 90-day mortality in patients who underwent RPD [9]. The ongoing integration of minimally invasive and specifically the robotic approach in pancreatic surgery led to the development of evidence-based consensus guidelines and recommendations [15, 28]. Two main key points were highlighted: [1] there is no evidence to suggest superiority of robotic approach in pancreatoduodenectomy, due to lack of prospective randomized trials, and [2] RPD should be utilized in high-volume centers for pancreatic and minimally invasive surgery that have surpassed the learning curve. The significance of patient volume and learning curve in RPD has been previously underlined and associated with improvement in patient outcomes. Adam et al. defined a minimum number of 22 minimally invasive pancreatoduodenectomies per year to correlate with the decrease in postoperative complications [29]. Similarly, passing the learning curve in RPD was associated with improved morbidity and mortality rates as well; the number of RPD cases that constitute the learning curve has been previously identified to be at least 40 cases as an individual surgeon up to 250 cases within a single institution [8, 11, 30, 31]. All participating centers in the PORTAL trial are considered high-volume centers in pancreatic surgery and MIS and have surpassed the learning curve in RPD; therefore, the PORTAL trial is characterized as a phase 3 trial.

This study was designed as a non-inferiority trial, and the rationale for this decision was based on the lack of robust retrospective data to demonstrate the superiority of RPD versus OPD, regarding functional recovery. The goal is to prospectively examine the outcomes in RPD and demonstrate non-inferiority of this approach compared to OPD. Furthermore, the decision to focus on RPD only and not include laparoscopic pancreatoduodenectomy patients was multifactorial: the robotic platform offers extra degrees of freedom and improved surgical ergonomics and was previously found to have an advantage compared to the laparoscopic approach [32]. Additionally, LEOPARD-2, a prospective randomized trial comparing LPD versus OPD, failed to demonstrate the superiority of LPD in functional recovery, but most importantly, the minimally invasive arm was associated with more complication-related deaths and the study was terminated early for safety purposes [6]. These results have revealed both the significance of the learning curve and surgical experience in MIS.

The number of enrolled patients in the PORTAL trial is 244 and was calculated taking under consideration three main protocol parameters: [1] conversion to open surgery in the RPD arm, which was defined as 10% based on historical institutional data [2]; intraoperative findings of occult abdominal metastatic disease; and [3] loss to follow-up, which in randomized controlled trials where included patients are closely monitored is approximately 3% [33]. The selection of eligible patients was limited to upfront resectable lesions without the involvement of major vessels. RPD with vascular resection or in patients with PDAC who previously underwent neoadjuvant are being performed in the participating institutions in increasing numbers, and recent reports have demonstrated their safety and feasibility [34, 35]. However, the technical challenges encountered in these patients would considerably affect the studied outcomes. All participating institutions are national referral centers and are considered very high volume in terms of open and robotic pancreatic surgery. Considering the number of patients that are being reviewed on a daily basis, we expect no delays in patient recruitment via an initial approach at the outpatient clinic preoperatively.

The primary endpoint of this study is functional recovery, which is a more objective evaluation of recovery and combines postoperative complications and patient-reported quality of life (QoL) outcomes. It is determined by four main parameters in the immediate postoperative period: sufficient pain control, nutritional competency, mobilization, and no evidence of ongoing inflammation [36]. Indeed, these criteria are of main interest in all patients and remain the focus of conversation during preoperative decision-making: when will the patient be able to resume his daily activities. Moreover, patient-reported outcomes are widely accepted and incorporated in patient care [37, 38]. Additionally, the concept of functional recovery is separate from inelastic variables, such as postoperative complications and/or postoperative length of hospital stay [39]; different healthcare systems around the world have different capacities for patient care management in the community [40]. However, postoperative complication rate and severity, and length of stay will be compared between RPD and OPD as secondary endpoints. The established EuroQoL Group 5-level EQ-5D questionnaire was utilized for the assessment of the quality of life [https://euroqol.org/eq-5d-instruments/]. Similar to previous randomized trials comparing minimally invasive and open surgery [41], patient blinding is being applied to reduce bias due to patient expectations and limit the effect of psychological factors associated with either of the two surgical approaches [42].

The second primary endpoint of this study is the timing of adjuvant chemotherapy initiation in patients with pancreatic adenocarcinoma, who represent the main bulk of pancreatoduodenectomies. The role of chemotherapy in PDAC has been long established, and different regimens have been identified to provide survival benefits [43, 44]. A recent retrospective study by Girgis et al. demonstrated that long-term oncological outcomes in patients with PDAC who undergo RPD are borderline superior compared to the open approach [45]. These results differentiate from a large study on cervical carcinoma that showed inferior overall survival in the minimally invasive group [46]. Thus far, no prospective data exist to support non-inferiority of RPD regarding oncological outcomes. The optimal time for chemotherapy treatment in the postoperative setting in PDAC has been identified to be no later than 12 weeks in order to achieve a combination effect of local and systemic treatments, yet most oncologists aim to start at 8 weeks [47]. Based on institutional data, we identified the percentage of patients who initiate adjuvant chemotherapy to be approximately 50% in OPD and 75% in RPD, and we aim to compare the two groups and assess for the superiority of RPD (64 patients in each arm). Furthermore, we will compare the recurrence-free survival and overall survival between the two groups, as well as the patterns of recurrence which are closely associated with long-term outcomes in PDAC [48].

Secondary endpoints of the PORTAL trial include perioperative and long-term outcomes. A recent meta-analysis [27] and smaller retrospective series within the past few years have demonstrated significantly lower overall complication rates in RPD compared to the open approach, as well as benefits in terms of estimated intraoperative blood loss. Additionally, retrospective data on RPD in PDAC patients have shown increased R0 resection rates and higher numbers of harvested lymph nodes [27, 49] and no difference in recurrence-free and overall survival [50]. The targeted analysis of PDAC patients within the PORTAL trial will be able to answer the question of long-term oncological outcomes in RPD. Moreover, a comprehensive cost analysis will include and compare operative charges, postoperative institutional expenses, and potential readmission costs to identify in detail the presence—or lack thereof—of the economic burden of robotics utilization in pancreatoduodenectomy [51].

In conclusion, the primary goal of the PORTAL trial is to study the short-term and long-term outcomes in RPD, compare them with the established OPD, and provide high-level evidence regarding the value of the robotic approach in pancreatoduodenectomy. Proof of non-inferiority may allow wider implementation of RPD in high-volume centers and engage for further studies focusing on more complex robotic pancreatic surgery. The goal is to publish the results of the trial in a high-impact peer-reviewed journal.

## Trial status

The reported study protocol for the PORTAL trial is the final version 1.4.1, dated May 26, 2020. Patient recruitment was initiated in September 2020 and is estimated to be completed by December 2022.

## Supplementary Information

**Additional file 1.** SPIRIT 2013 Checklist: Recommended items to address in a clinical trial protocol and related documents*.

## Data Availability

The final trial dataset will be available to all investigators from the participating institutions.

## Abbreviations

RPD: Robotic pancreatoduodenectomy
OPD: Open pancreatoduodenectomy
PDAC: Pancreatic ductal adenocarcinoma
MIS: Minimally invasive surgery
LPD: Laparoscopic pancreatoduodenectomy
CT: Computed tomography
MRI: Magnetic resonance imaging
CA19-9: Cancer antigen 19-9
NRS-2002: Nutritional risk screening
POPF: Postoperative pancreatic fistula
BMI: Body mass index
SMV: Superior mesenteric vein
PV: Portal vein
GDA: Gastroduodenal artery
SMA: Superior mesenteric artery
PJ: Pancreaticojejunostomy
HJ: Hepaticojejunostomy
GJ: Gastrojejunostomy
NGT: Nasogastric tube
POD: Postoperative day
RFS: Recurrence-free survival
OS: Overall survival
PD: Pancreatic duct
